# Genetic Heterogeneity of Residual Variance for Foot Score Traits in American Angus Cattle

**DOI:** 10.1111/jbg.12949

**Published:** 2025-06-21

**Authors:** Sabrina T. Amorim, Kelli J. Retallick, André Garcia, Noelia Ibañez‐Escriche, Gota Morota

**Affiliations:** ^1^ School of Animal Sciences Virginia Tech Virginia USA; ^2^ Angus Genetics Inc. American Angus Association Saint Joseph Missouri USA; ^3^ Institute for Animal Science and Technology Universitat Politècnica de València València Spain; ^4^ Laboratory of Biometry and Bioinformatics, Department of Agricultural and Environmental Biology, Graduate School of Agricultural and Life Sciences The University of Tokyo Tokyo Japan

**Keywords:** beef cattle, claw set, foot angle, foot scores, genetic heterogeneity of residual variance

## Abstract

Foot conformation is one of the main breeding goals in recent beef cattle breeding programs because it directly affects productivity, economic losses, animal welfare and longevity. Genetic heterogeneity of residual variance can be used to improve production uniformity in animal breeding programs because recent studies have shown that residual variance is partially under genetic control, allowing reduction of variability through selection. Despite being an important breeding goal, research on genetic heterogeneity of residual variance for conformation traits, such as foot angle and foot claw, is still scarce in livestock species. The objectives of our study were (1) to investigate the extent of genetic heterogeneity of residual variances on two conformation traits: foot angle (FA) and claw set (CS) in Angus cattle using genetic homogeneity (M1) and two genetic heterogeneity of residual variance models, including a double hierarchical generalised linear model (DHGLM, M2) and a genetically structured environmental variance model (M3). Genetic parameters for means and residual variances were estimated using M2 and M3. The dataset included 45,667 phenotypic records for FA and CS (scores from 1 to 9 with 5 being ideal) of American Angus cattle recorded from 2009 to 2021. M1 and M2 were fitted using average information restricted maximum likelihood, and M3 was fitted using Markov chain Monte Carlo. Heritability estimates for the means of FA (0.19 ± 0.007 for M1, 0.11 ± 0.005 for M2 and 0.09 ± 0.003) and CS (0.16 ± 0.005 for M1, 0.10 ± 0.004 for M2 and 0.08 ± 0.03) were within the range reported in the literature, but M2 and M3 estimates were lower than M1. Genetic heterogeneity of residual variance was assessed using three parameters: heritability for residual variance, genetic coefficient of variation, and correlation between mean and residual variance. Although heritability estimates for residual variance in M2 were low (0.08 for FA and 0.001 for CS), our results suggest that residual variance is partially under genetic control. The genetic coefficients of variation estimates were 0.08 (M2) and 0.06 (M3) for FA, and 0.06 (M2) and 0.02 (M3) for CS, indicating that selection on the trait mean would also change the residual variance. Our results for FA and CS showed moderate positive genetic correlations in M2 (0.52 for FA and 0.41 for CS) and M3 (0.35 for FA and 0.33 for CS) between mean and residual variance. Positive correlations may limit the response to selection unless other breeding strategies, such as selection indices, are used. FA and CS are promising traits for uniformity or resilience indicators because they are phenotypes that can be collected throughout the production cycle using traditional or digital data recording systems. Our results demonstrate the potential to modulate variability through breeding strategies and present an opportunity to evaluate the uniformity of foot score traits in beef cattle.

## 1 Introduction

Hoof structure is a major concern in global dairy and beef cattle production and is critical for optimal animal performance, health and longevity (Giess et al. 2021). Identifying structural disorders is critical to animal welfare because they can lead to lameness, which can result in economic losses due to reduced production, fertility issues and veterinary costs (Van Dorp et al. 2004). In addition, Oliveira et al. (2020) reported that approximately 6% of cows are culled due to structural problems, which has a direct impact on the longevity of the animal.

Traditionally, beef cattle breeding programs have focused on production and reproduction traits because these are the primary profit drivers for the industry. However, the North American and Australian Angus Associations have defined foot conformation (structural soundness) as one of the top three breeding goals (Bell et al. 2019; Santos et al. 2019). Selection for hoof structure can be performed using the foot scoring system, with foot angle (FA) and claw set (CS) being the key indicators of structural disorders. Recently, efforts from both associations have spurred interest in the genetic evaluation of foot score traits (Alvarenga et al. 2023), drawing attention to the evaluation of animals in different environments and the potential re‐ranking of breeding candidates for specific environmental conditions due to genotype‐by‐environment interactions (Hayes et al. 2016) or microenvironmental sensitivity.

Uniformity of production refers to the goal of reducing trait variability in populations to achieve more consistent and predictable outcomes. This is particularly important for traits that affect productivity, economic efficiency, animal welfare and product quality, and it has recently become an important breeding goal in several livestock species (Iung et al. 2020). In addition, lack of uniformity has been associated with economic losses in many species (Abiola et al. 2008; Mee 2008; Milligan et al. 2002; Pardo et al. 2013; Wilson 1991). Breeding strategies, such as mating systems and selection play an important role in influencing trait variability within animal populations. Recently, numerous studies have shown that residual variance may be under genetic control (Hill and Mulder 2010; Iung et al. 2017), which can potentially be used to enhance the uniformity of foot score traits. Genetic control of uniformity, also referred to as genetic heterogeneity of residual variance or genetic variance in microenvironmental sensitivity, can provide insight into how genotypes respond differently to unknown microenvironmental factors (Ehsaninia et al. 2019; Falconer and Mackay 1996; Hill and Mulder 2010; Iung et al. 2020).

Several approaches have been developed to study genetic heterogeneity of residual variance (Felleki et al. 2012; Mulder et al. 2009; Rönnegård et al. 2010; SanCristobal‐Gaudy et al. 1998), providing solutions for response variables at the level of mean and residual variance. However, there is still a lack of studies on genetic heterogeneity of residual variance in beef cattle (Iung et al. 2017; Neves et al. 2012, 2011), and no reports on structural traits such as FA and CS. Therefore, the objectives of our study were (1) to investigate the extent of heterogeneity in residual variance for foot score traits in American Angus cattle and (2) to compare the results of pedigree‐based REML and Bayesian approaches.

## 2 Material and Methods

### 2.1 Data

#### 2.1.1 Ethics Statement

Institutional Animal Care and Use Committee approval was not required for this study because the data were obtained from an existing American Angus Association database.

### 2.2 Data

**FIGURE 1:**
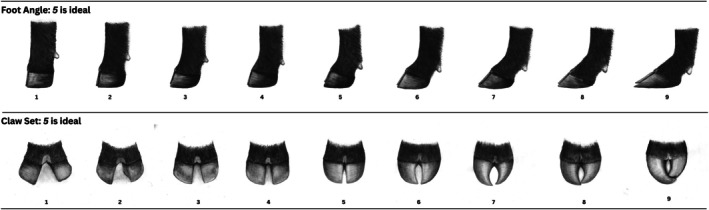
Foot score guidelines developed by the American Angus Association. Picture. American Angus Association (Retallick 2019).

The dataset was provided by the American Angus Association and included phenotypic information for two foot score traits: FA and CS. The foot scoring system, developed by the American Angus Association, is a subjective measurement ranging from 1 to 9, as shown in Figure 1. A score of five is optimal for both FA and CS.

For FA, an animal with a score of five would have a 45° angle in the pastern along with a satisfactory heel depth. For CS, a score of five reflects claws that are straight and even (symmetrical). Phenotypes were collected at the yearling stage (one year of age) or older. It is recommended that repeated measurements be taken prior to hoof trimming. Additionally, the association advises that FA and CS be recorded on the worst foot when contemporary animals are handled, with a single handler recording all scores within a contemporary group (Retallick 2019).

Contemporary groups (CG) consisted of a concatenation of the following systematic effects: weaning and yearling herd, lot identification, measurement date, management code (e.g., creep feeding system), diet type (i.e., low concentrate diet < 50%, high concentrate diet > 50%, or pasture), herd, and date of foot score collection.

The phenotypic datasets included data from registered purebred Angus cattle. A total of 190,140 phenotypes (118,828 animals recorded from December 2008 to October 2022) from the USA were available for analysis. The datasets were edited to maintain sufficient variation within each level of CG (at least 150 records and two foot score categories) and age ranging from 321 to 7796 days. Due to software limitations and to maintain consistency between the models studied, only a single record per animal was included in the analysis.

### 2.3 Variance Components Estimation

Variance components for the mean and residual variance of FA and CS were estimated using three models. The first model is a homoscedastic model (M1), which follows a traditional additive genetic framework in which the residual variance is homogeneous. The subsequent models, M2 and M3, jointly estimate parameters for the mean and residual variance.

### 2.4 Homoscedastic Residual Variance Model (M1)

The first model was a standard univariate animal model for FA and CS, which is a classical additive genetic model including the assumption of homogeneity of residual variance. For both traits, M1 was

y=Xb+Za+e

where y is a vector of phenotypes (FA or CS); X is an incidence matrix of systematic effects; b is a vector of systematic effects (CG as previously defined, and age of animal as a covariate); Z is an incidence matrix relating individuals to phenotypic records; a is the additive genetic effect with $a\sim N\left(0,A\sigma_{a}^{2}\right)$, where A is the numerator relationship matrix (traced back up to 3 generations) and $\sigma_{a}^{2}$ is the additive genetic variance; and e is a vector of residuals, assuming that $e\sim N\left(0,{I\sigma }_{e}^{2}\right)$, where I is an identity matrix and $\sigma_{e}^{2}$ is the residual variance. M1 was solved using the Average Information Restricted Maximum Likelihood (AI‐REML) module of the DMU package (Madsen et al. 2013).

### 2.5 Heterogeneous Residual Variance Models

#### 2.5.1 Double Hierarchical Generalised Linear Model (M2)

A double hierarchical generalised linear model (DHGLM) is an extension of generalised linear models (Lee and Nelder 1996), which can handle non‐normal data by assuming a gamma distribution for the squared residuals (Rönnegård et al. 2010). It is an iterative approach that addresses the non‐normality issue by focusing on modelling the squared residuals rather than the raw data. The algorithm iterates using a bivariate analysis by fitting the mean (or the trait) and residual variance *as follows* (Felleki et al. 2012):

$$\left[\begin{array}{c} y\\ \psi \end{array}\right]=\left[\begin{array}{cc} X & 0\\ 0 & X_{V} \end{array}\right]\left[\begin{array}{c} b\\ b_{v} \end{array}\right]+\left[\begin{array}{cc} Z & 0\\ 0 & Z_{V} \end{array}\right]\left[\begin{array}{c} a\\ a_{v} \end{array}\right]+\left[\begin{array}{c} e\\ e_{v} \end{array}\right]$$

where y and ψ are vectors of response variables for the trait (FA or CS) and residual variance, respectively. ψ is defined as

$$\psi_{i}=\log\left({\hat{\sigma }}_{e_{i}}^{2}\right)+\left(\left\{\left[\;{\hat{e}}_{i}^{2}/\left(1-h_{i}\right)\right]-{\hat{\sigma }}_{e_{i}}^{2}\right\}/{\hat{\sigma }}_{e_{i}}^{2}\right)$$

where ${\hat{\sigma }}_{e_{i}}^{2}$ is the predicted residual variance for observation i from the previous iteration; ${\hat{e}}_{i}^{2}$ is individual‐specific environmental variance; $h_{i}$ is the leverage defined as the $i^{\mathrm{th}}$ diagonal element of the hat matrix H corresponding to the $i^{\mathrm{th}}$ individual (Hoaglin and Welsch 1978), assessing how much variability in the response variables is explained by the fixed and random effects; Xb and $\;X_{V}\;b_{V}$ are fixed effects for the mean and residual variance parts, respectively; Zaand $\;Z_{V}\;a_{V}$are the additive genetic effects for the mean and residual variance parts, respectively; and e and $e_{v}$ are residuals for the mean and residual variance parts, respectively. The other effects in the model are the same as those described above for M1. It was also assumed that

$$\left[\begin{array}{c} a\\ a_{v} \end{array}\right]\sim N\left(0,\left[\begin{array}{cc} \sigma_{a}^{2} & \sigma_{a,a_{v}}\\ \sigma_{a,a_{v}} & \sigma_{a_{v}}^{2} \end{array}\right]⨂A\;\right)$$

where ⨂ denotes the Kronecker product.

The residuals e and $e_{v}$ were independent and followed a normal distribution,

$$\left[\begin{array}{c} e\\ e_{v} \end{array}\right]\sim N\left(0,\left[\begin{array}{cc} W^{-1}\sigma_{e}^{2} & 0\\ 0 & W_{v}^{-1}\sigma_{e_{v}}^{2} \end{array}\right]⨂I\;\right)$$

where W and $W_{v}$ are diagonal matrices defined as $W=\mathrm{diag}\left({\hat{\psi }}^{-1}\right)$ and $W_{v}=\mathrm{diag}\left(\frac{1-h}{2}\right)$, respectively, and are the residual variance per observation, since $\sigma_{e}^{2}$ and $\sigma_{e_{v}}^{2}$ are scaling variances that are expected to be equal to 1. $\hat{\psi }$ is the updated estimate of the residual variance for each observation at a given iteration, and h refers to the vector of leverage values for all individuals in the dataset. Estimation of the variance components was performed using the iterative AI‐REML algorithm in the DMU software (Madsen et al. 2013). The parameters, ψ,W and $W_{v}$, were updated at each iteration until the algorithm converged. Convergence was assumed when the relative difference in the estimates of the variance components between iterations was less than $10^{-6}$.

### 2.6 Genetically Structured Variance Model (M3)

The quantitative genetic model for M3 is the exponential model (SanCristobal‐Gaudy et al. 1998), which is an extension of M1. The residual variance is assumed to be heterogeneous and partially under genetic control,

$$y_{i}=x_{i}b+z_{i}a+e^{\frac{1}{2}\left(x_{i}b^{*}+z_{i}a^{*}\right)}\varepsilon_{i}$$

where $y_{i}$ is FA or CS of the $i^{\mathrm{th}}$individual; * indicates parameters associated with the residual variance; b and $b^{*}$ are the vectors of systematic effects; a and $a^{*}$ are the vectors of direct genetic effects; $x_{i}$, $z_{i}$, are the same as previously described for M1 and M2; and $\varepsilon_{i}\sim N\left(0,1\right)$ is the residual of animal i.

The genetic effects a and $a^{*}$ are jointly distributed and assumed to be Gaussian,

$$\left[\begin{array}{c} a\\ a^{*} \end{array}\right]\sim N\left(\left[\begin{array}{c} 0\\ 0 \end{array}\right],\left[\begin{array}{cc} \sigma_{a}^{2} & \rho \sigma_{a}\sigma_{a^{*}}\\ \rho \sigma_{a}\sigma_{a^{*}} & \sigma_{a^{*}}^{2} \end{array}\right]⨂A\;\right)$$

where A is the numerator relationship matrix; $\sigma_{a}^{2}$ is the additive genetic variance; $\sigma_{a^{*}}^{2}$ is the additive genetic variance for the residual variance; ρ is the coefficient of correlation between $\sigma_{a}^{2}$ and $\sigma_{a^{*}}^{2}$; and ⨂ denotes the Kronecker product.

M3 was fit using the GSEVM program (Ibáñez‐Escriche et al. 2010). The results of M3 were obtained by averaging the results of Markov chain Monte Carlo samples after 1,000,000 iterations with a burn‐in period of 100,000 iterations. The Geweke criterion (Geweke 1991) was used to diagnose chains at a 5% significance level using the boa package (Smith 2007) in R (R Core Team 2024). Inferences were based on probabilities obtained from marginal posterior distributions of the parameters or their combinations. The means of the marginal posterior distributions were taken as estimates, and $\sigma_{a^{*}}^{2}$ was compared to $\sigma_{a_{v}}^{2}$ from M2.

The heritability estimates of the means for FA and CS under model M3 are not fixed because they depend on the residual variance, which is influenced by varying environmental effects (represented by $b^{*}$) (Formoso‐Rafferty et al. 2017; Ibáñez‐Escriche et al. 2008). The conditional variance of the observed trait, $y_{i}$, given the systematic effects b and $b^{*}$, is represented as follows:

Varyib,b*=σa2+expXb*i+σa*22

Therefore, heritability estimates ($h_{i}^{2}$) obtained as the mean for each combination of levels of systematic effects ${\left(Xb^{*}\right)}_{i}$ as follows (Ros et al. 2004; Sorensen and Waagepetersen 2003):

$$h_{i}^{2}=\frac{\sigma_{a}^{2}}{\sigma_{a}^{2}+\exp\left({\left(Xb^{*}\right)}_{i}+\frac{\sigma_{a*}^{2}}{2}\right)}$$

### 2.7 Genetic Parameters Associated With Residual Variance

Three genetic parameters were calculated to interpret the genetic heterogeneity of residual variance in our study: heritability of residual variance ($h_{v}^{2}$), genetic coefficient of variation of residual variance $\left({\mathrm{GCV}}_{E}\right)$, and genetic correlation between mean and residual variance ($r_{\mathrm{mv}}$).

The $h_{v}^{2}$ can be used to evaluate the accuracy of the estimated breeding values for the residual variance. $h_{v}^{2}$ was estimated as defined by (Mulder et al. 2007):

$$h_{v}^{2}=\frac{\sigma_{a_{v,\mathrm{add}}}^{2}}{\left(2\sigma_{p}^{4}+3\sigma_{a_{v,\mathrm{add}}}^{2}\right)}$$

where $\sigma_{a_{v,\mathrm{add}}}^{2}$ is the additive genetic variance estimated for the residual variance on the additive scale and $\sigma_{p}^{4}$ is the squared phenotypic variance. Here, $\sigma_{a_{v,\mathrm{add}}}^{2}$ and the standard error of $h_{v}^{2}$ were obtained following Mulder et al. (2016).

${\mathrm{GCV}}_{E}$ infers how much the residual variance could be changed by selection (Mulder et al. 2016) and was calculated as:

$$\mathrm{GC}V_{E}=\frac{\sigma_{a_{v,\mathrm{add}}}}{\bar{{\bar{\sigma }}_{\hat{e}}^{2}}}$$

where $\sigma_{a_{v,\mathrm{add}}}$ is the standard deviation of the additive genetic variance for the residual variance on the additive scale. $\mathrm{GC}V_{E}$ in M3 was calculated as described in Rönnegard et al. (2013).

The genetic correlation between mean and residual variance ($r_{\mathrm{mv}}$) was estimated as:

$$r_{\mathrm{mv}}=\frac{\sigma_{a,a_{v}}}{\sqrt{\sigma_{a}^{2}\sigma_{a_{v}}^{2}}}$$

where $\sigma_{a,a_{v}}$, $\sigma_{a}^{2}$, and $\sigma_{a_{v}}^{2}$ were previously defined for M2.

## 3 Results

**FIGURE 2:**
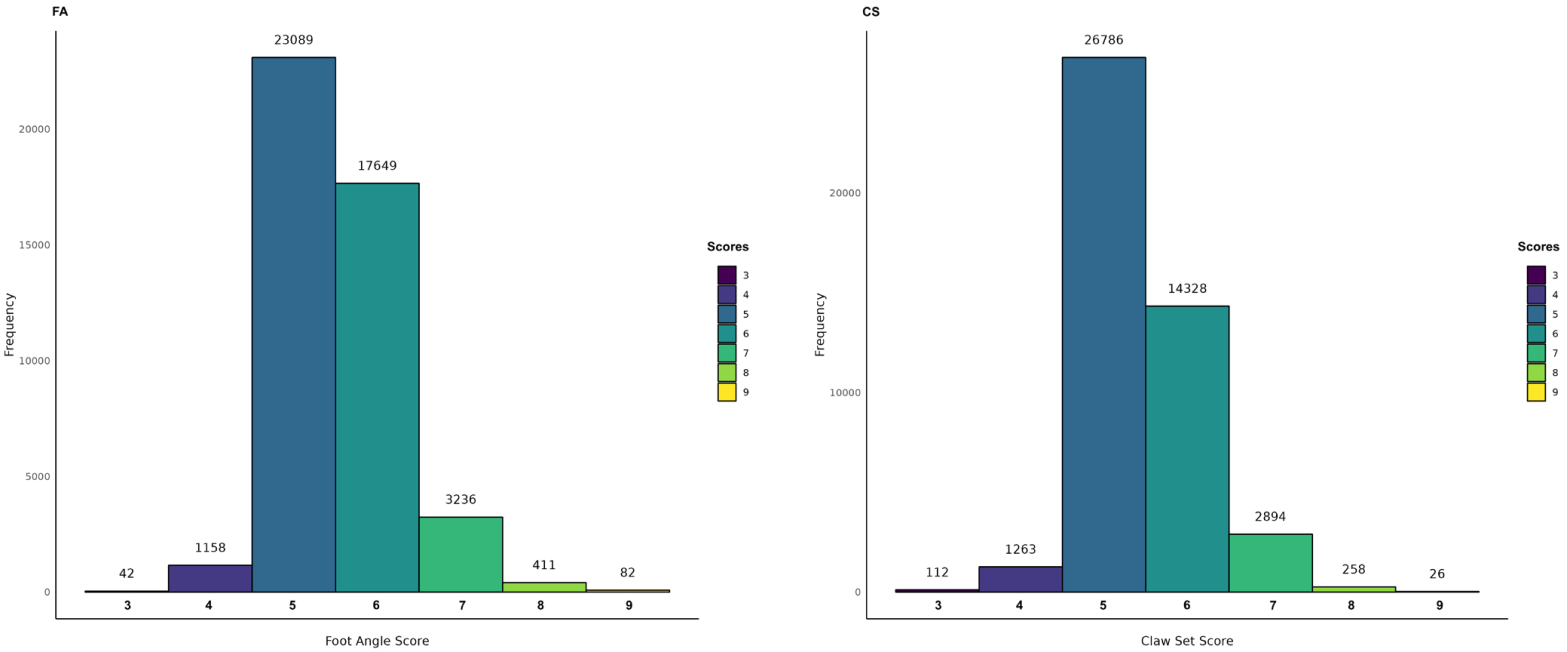
Distribution of foot angle (FA) and claw set (CS) scores after data cleaning. [Colour figure can be viewed at wileyonlinelibrary.com]

After quality control, 45,667 records (45,667 animals recorded from October 2009 to November 2021) remained for further analysis. Tables 1 and 2 present the data structure and descriptive statistics. The distribution of foot scores after data cleaning is shown in Figure 2. The posterior estimates of systematic effects on residual variances in M3 were 0.049 for CG and 0.016 for age in FA, and 0.087 for CG and 0.027 for age in CS.

**TABLE 1:** Data structure of foot score traits after data cleaning.

Data structure	FOOT angle and claw set
Number of records	45,667
Number of animals in pedigree	80,808
Number of CG	1111
Number of sires	3069
Number of dams	32,072
Average number of progeny per Sire	16.81
Average number of animals per contemporary groups	46.46

Abbreviation: CG, contemporary groups.

**TABLE 2:** Descriptive statistics of foot score traits.

Descriptive statistics	Foot angle	Claw set
Minimum	3.00	3.00
Maximum	9.00	9.00
Mean ± SD	5.45 ± 0.71	5.55 ± 0.73
Skewness	0.77	0.78
Kurtosis	1.38	1.31

Abbreviation: SD, standard deviation.

### 3.1 Homoscedastic Residual Variance Model (M1)

**FIGURE 3:**
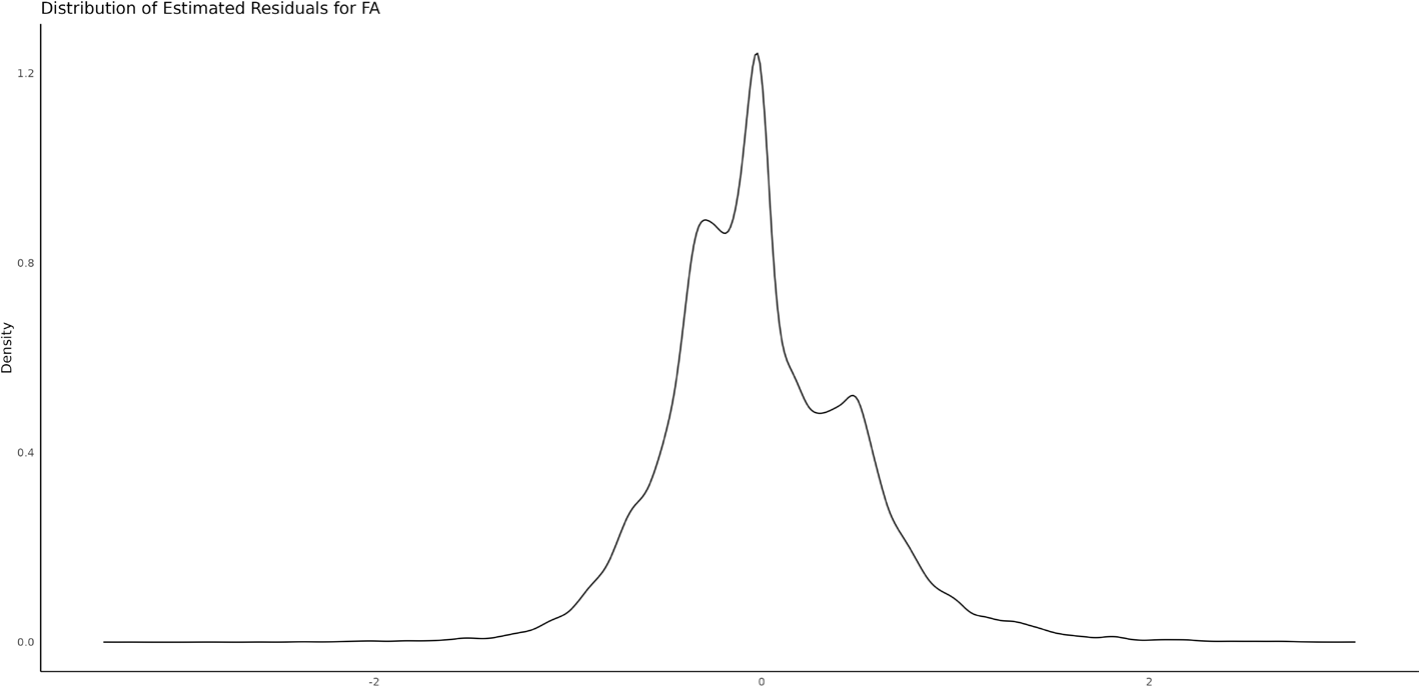
Estimated residual distribution for foot angle using M1.

**FIGURE 4:**
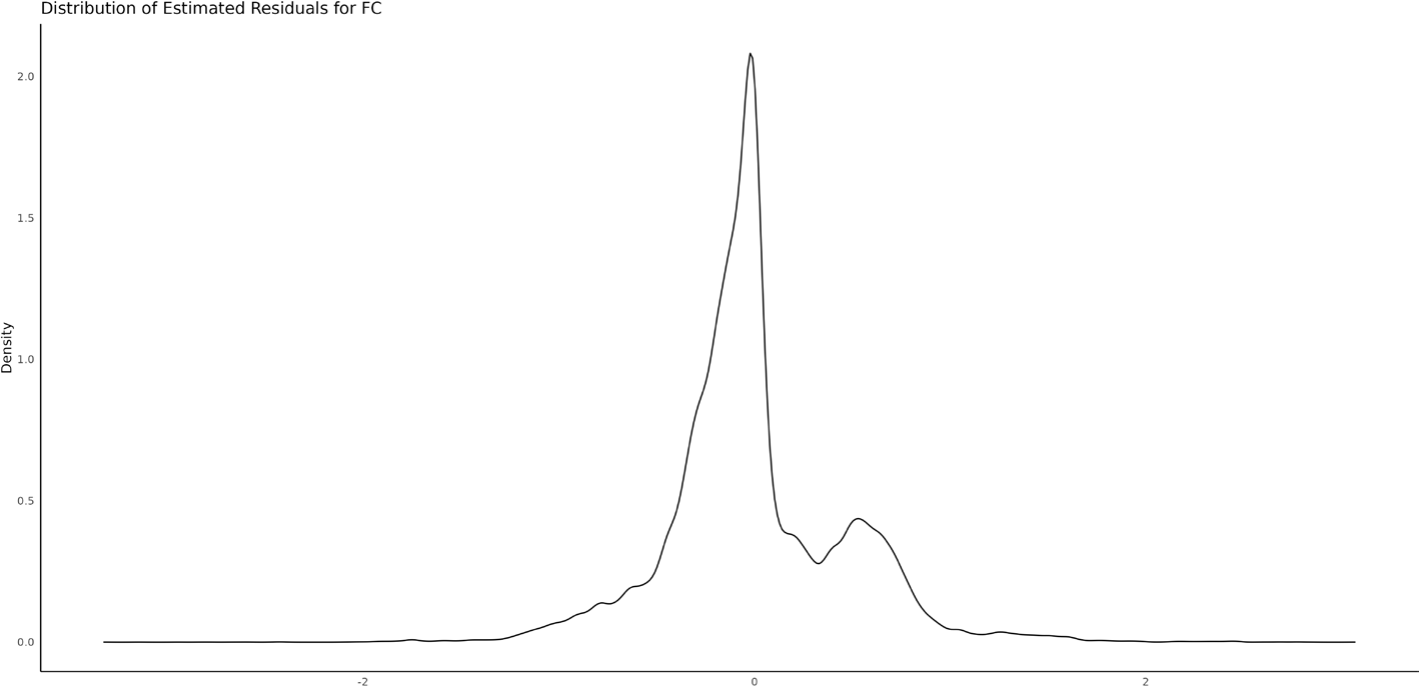
Estimated residual distribution for claw set using M1.

Estimates of variance components and genetic parameters obtained from the three models for FA and CS are presented in Table 3. Heritability estimates were low, ranging from 0.19 (± 0.007) for FA to 0.16 (± 0.005) for CS. The estimated residual distributions are presented in Figures 3 and 4.

**TABLE 3:** Variance component estimates and genetic parameters for mean and residual variance for FA and CS using three different models.

Trait	Model	$\sigma_{a}^{2}$	$\sigma_{e}^{2}$	$\sigma_{\mathrm{av}}^{2}$ ^b^	$h^{2}$	$h_{v}^{2}$	${\mathrm{GCV}}_{E}$	$r_{\mathrm{mv}}$
FA	M1	0.07 $\left(3.00\times 10^{-3}\right)$	0.30 $\left(3.10\times 10^{-3}\right)$	—	0.19 $\left(7.00\times 10^{-3}\right)$	—	—	—
	M2	0.04 ($1.8\times 10^{-3}$)	0.31 (0.04)	$7.85\times 10^{-4}$ $\;\left(0.27\times 10^{-4}\right)$	0.11 (0.005)	0.08 $\left(7.00\times 10^{-3}\right)$	0.08 ($4.00\times 10^{-3}$)	0.52 (0.14)
	M3^a^	0.05 $\left(0.19\times 10^{-2}\right)$	—	$4.35\times 10^{-4}$ ($0.14\times 10^{-4}$)	0.09 ($3.00\times 10^{-3}$)	—	0.06 $\left(8.00\times 10^{-3}\right)$	0.35 (0.05)
CS	M1	0.04 $\left(4.00\times 10^{-3}\right)$	0.24 $\left(4.00\times 10^{-3}\right)$	—	0.16 $\left(5.00\times 10^{-3}\right)$	—	—	—
	M2	0.03 ($1.00\times 10^{-3}$)	0.31 $\left(9.00\times 10^{-3}\right)$	$3.57\times 10^{-4}$ $\left(0.11\times 10^{-3}\right)$	0.10 $\left(4.00\times 10^{-3}\right)$	$1.00\times 10^{-3}$ ($0.46\times 10^{-3}$)	0.06 $\left(3.00\times 10^{-3}\right)$	0.41 (0.12)
	M3^a^	0.02 $\left(0.12\times 10^{-3}\right)$	—	$7.28\times 10^{-4}$ $\;\left(0.23\times 10^{-3}\right)$	0.08 (0.03)	—	0.02 ($0.14\times 10^{-3}$)	0.33 (0.09)

*Note:* $\sigma_{a}^{2}$ and $\sigma_{\mathrm{av}}^{2}$ are the additive genetic variance for the means and residual variances. $\sigma_{e}^{2}$ is the residual variance. $h^{2}$ and $h_{v}^{2}$ are the heritability estimates for the means and residual variances. ${\mathrm{GCV}}_{E}$ is the genetic coefficient of variation for the residual variance; $r_{\mathrm{mv}}$ is the genetic correlation between the estimated breeding values of the means and residual variances; standard errors are given in parentheses.

Abbreviations: CS, claw set; FA, foot angle; M1, homoscedastic residual variance model; M2, heteroscedastic model (DHGLM); M3, heteroscedastic model (structured model).

^a^ Mean and SE (in parentheses) of the marginal posterior distribution.

^b^ For M3, $\sigma_{\mathrm{av}}^{2}$ represents $\sigma_{a^{*}}^{2}$.

### 3.2 Heterogeneous Residual Variance Models

The additive genetic variance ($\sigma_{a}^{2}$) for the mean in M2 was similar but slightly lower than in M1 for FA (0.07 in M1, 0.04 in M2, and 0.05 in M3) and a similar pattern was observed for CS (0.04 in M1, 0.03 in M2, and 0.02 in M3). There was a decrease in the estimates of $\sigma_{a}^{2}$ in M2 and M3, therefore, $\sigma_{a}^{2}$in M1 presented the highest among the three models in this study. Heritability estimates ($h^{2}$) for the mean in M2 and M3 were also lower for FA (0.11 in M2 and 0.09 in M3) and CS (0.10 in M2 and 0.08 in M3) than in M1.

We estimated the parameters influencing the genetic control of the residual variance with M2 and M3. In M2, the genetic variance for the residual variance $\left(\sigma_{\mathrm{av}}^{2}\right)$ was $7.85\times 10^{-4}$for FA and $3.57\times 10^{-4}$ for CS. In M3, the posterior means of $\sigma_{a^{*}}^{2}$ were $4.35\times 10^{-4}$ for FA and $7.28\times 10^{-4}$ for CS.

The heritability estimates of the residual variance $\left(h_{v}^{2}\right)$ were obtained using M2. Low estimates of $h_{v}^{2}$ (< 0.1) were obtained for both traits, ranging from 0.08 ± 0.007 for FA to 0.001 ± 0.0005 for CS. Estimates of $\mathrm{GC}V_{E}$ were low for FA and CS in M2, ranging from 0.08 for FA to 0.06 for CS. The estimates of $\mathrm{GC}V_{E}$ in M3 were 25% lower (0.06) for FA than that of M2 and ~ 66% lower (0.02) for CS than that of M2.

Finally, the genetic correlation between the mean and residual variance ($r_{\mathrm{mv}}$) was estimated using M2 and M3. In both models, moderate positive correlations were found, and the highest values of $r_{\mathrm{mv}}$ were observed in M2, ranging from 0.52 for FA to 0.41 for CS. Lower positive correlations were obtained in M3 ranging from 0.35 to 0.33 for FA and CS, respectively.

## 4 Discussion

### 4.1 Genetic Parameters for the Mean

Hoof structure is crucial in beef cattle production, as poor feet and legs are among the most common reasons for culling beef cattle from a herd. Culling animals can be costly, especially in situations where raising or rearing an animal does not generate sufficient income to cover these production costs (Giess et al. 2021). Despite the economic importance of foot score traits in beef cattle, research on these traits remains scarce (Alvarenga et al. 2023; Giess et al. 2021; Jeyaruban et al. 2012; Saad et al. 2021; Wang et al. 2017).

First, it should be noted that some studies may report foot scores for both front and rear legs. However, it has been shown that front and rear leg scores are highly genetically correlated (~ 0.88 for FA and ~ 0.75 for CS) (Giess et al. 2021), meaning that only one record in the front or rear leg could be recorded and used to select individuals based on foot scores. Based on this high genetic correlation, the data analysed in the current study collected one record (front or rear leg) from each animal instead of two records to optimise phenotype collection (Retallick 2019). The estimates of $h^{2}$ for FA obtained in this study (0.19 in M1, 0.11 in M2, and 0.08 in M3) were lower than those observed by Jeyaruban et al. (2012), who reported $h^{2}$ estimates of 0.32 and 0.29 for front and rear feet angle in Australian Angus. Our results were also lower than those reported by Wang et al. (2017) in the American Angus population, with animals ranging in age from 320 to 810 days (0.37) and by Saad et al. (2021) in a multibreed population with animals ranging in age from 8 months to 16 years (0.26). More recently, Alvarenga et al. (2023), in a study of foot score traits in American and Australian Angus cattle reported slightly higher $h^{2}$ estimates for FA in the US population (0.22) compared to estimates in M1. However, it is important to note that the dataset used in this study is a subset of the US dataset in Alvarenga et al. (2023), so lower $h^{2}$ estimates were expected in M1. Our estimates of $h^{2}$ for FA in M1 were higher than those found by Giess et al. (2021) for front and rear hoof angles measured at 1 to 2 years of age for bulls and 1 to 18 years of age for cows in Red Angus (0.18 and 0.17, respectively). The estimate of $h^{2}$ for FA in M2 (0.11) and M3 (0.09) in our study was lower than all other estimates reported in the literature.

For CS, the range of heritability estimates in the different studies was between 0.08 and 0.33. Our estimates for CS were higher than those of Giess et al. (2021) for front (0.08) and rear (0.15) CS in M1, the same (0.10) compared to M2, and the same (0.08) for the front CS in M3. Compared to the other studies, our estimates in M1, M2, and M3 were lower than those of Jeyaruban et al. (2012), who evaluated CS using only single records from animals younger than 750 days of age (0.33) and Saad et al. (2021) (0.29), who worked with a dataset of 4333 single records per animal and repeated records per animal (865 repeated records for FA and 864 for CS), for a total of 12,179 observations in their multi‐breed population of Black Angus, Red Angus, South Devon, British, Simmental, Charolais, Gelbvieh, Continental breeds, and others. In American Angus, Wang et al. (2017) used a subset of the dataset used in Alvarenga et al. (2023) (phenotypes collected up to 2016) and reported $h^{2}$for CS of 0.25, which was higher than the recent work by Alvarenga et al. (2023) (0.21). Another possible reason why our heritability estimates were lower than those of the aforementioned studies is the presence of scores 3 and 4 in the dataset. Previous studies have observed the same trend of decreasing heritability estimates when these scores (< 5) are included, which is why some studies (Alvarenga et al. 2023; Saad et al. 2021; Wang et al. 2017) chose to remove these phenotypes for further analysis. For example, Wang et al. (2017) reported that analysing FA and CS using scores ≥ 5 resulted in higher heritability estimates compared to including scores < 5. From a practical perspective, excluding lower scores simplifies the interpretation of expected progeny differences because lower expected progeny differences are more easily recognised as favourable by producers. In addition, we only used pedigree information in the analysis. However, the lack of representation in the dataset for FA and CS is likely due not only to the use of pedigree data, but also to the filtering process used to create sufficiently large CG. By excluding smaller herds to create a larger CG, a portion of the national herd may be left out, reducing the overall representativeness.

### 4.2 Genetic Variances and Parameters for the Variance

In general, animals are born with good hoof structure. As they grow, the locomotor system is influenced by genetic factors that may or may not be expressed at different stages of the animal's life, which can alter its posture. Additionally, environmental factors, such as overfeeding, food and water availability, and the topography of the grazing environment, can have lasting effects on bones and joints (Boelling and Pollott 1998; Langova et al. 2020; Tranter and Morris 1992).

To our knowledge, this is the first study to report the extent of genetic control of residual variance for FA and CS in beef cattle and therefore the discussion for these parameters will focus on other similar conformation‐related traits in chicken and cattle. Our results show the presence of additive genetic variances for the mean ($\sigma_{a}^{2}$) of FA and CS and their variances ($\sigma_{\mathrm{av}}^{2}$and $\sigma_{a^{*}}^{2}$).

Estimates of heritability of residual variance ($h_{v}^{2}$) were low, ranging from 0.08 (± 0.007) for FA to 0.001 (± 0.0005) for CS in M2. These estimates are within the range reported by Hill and Mulder (2010) and Iung et al. (2020) (between 0.05 and < 0.1). Wolc et al. (2009) estimated genetic parameters for the residual variance of conformation scores in chickens (where 1 was considered poor conformation and 5 was excellent conformation). The authors reported $h_{v}^{2}$ estimates for conformation scores of 0.03 (± 0.01) for females and 0.02 (± 0.004) for broilers. Similarly, in Nellore cattle, Neves et al. (2011) evaluated conformation scores (scale of 1 to 5) at weaning and yearling and reported 0.01 (± 0.004) for conformation score at weaning and 0.01 (± 0.002) for conformation score at yearling. These estimates were lower than those reported by Wolc et al. (2009).

Mulder et al. (2007) pointed out that large amounts of information are needed to accurately predict $h_{v}^{2}$. Our low estimates for $h_{v}^{2}$ compared to other studies in the literature for conformation‐related traits support this statement. Based on the estimated ${\mathrm{GCV}}_{E}$ values obtained from M2 and M3, the presence of a possible opportunity for genetic change in FA and CS through selection is suggested. The concept of ${\mathrm{GCV}}_{E}$ (also referred to as evolvability) was originally introduced by Houle (1992) as a measure of the genetic standard deviation relative to the mean of a trait, which is crucial for predicting the ability of a population to respond to selection. Our results indicate that a change of 1 genetic standard deviation would change the residual variance of FA by ~ 8% in M2 and ~ 6% in M3. For CS, a change in residual variance of ~ 6% in M2 and ~ 2% in M3 is expected. The ${\mathrm{GCV}}_{E}$ values obtained are lower than the range reported for conformation traits in chickens and cattle (0.15 to 0.31) (Neves et al. 2011; Wolc et al. 2009), but within the range of ${\mathrm{GCV}}_{E}$ reported for livestock and aquaculture species (0–0.86) (Hill and Mulder 2010; Iung et al. 2020).

The genetic correlation between mean and residual variance is represented by $r_{\mathrm{mv}}$. M2 showed the highest correlations of 0.52 for FA and 0.41 for CS. The range for conformation‐related traits in the literature is 0.15 to 0.40 (Neves et al. 2011; Wolc et al. 2009), which is within the range reported for various traits in cattle (−0.20 to 0.89) (Iung et al. 2020). The observed moderate $r_{\mathrm{mv}}$ values may also be related to pleiotropic effects, as most of the alleles of the mean of a trait may also act on its variance (Gavrilets and Hastings 1994). On the other hand, $r_{\mathrm{mv}}$ estimates were positive but lower in M3 than in M2, where the corresponding $r_{\mathrm{mv}}$ was 0.35 for FA and 0.33 for CS. Combined with $h_{v}^{2}$, the genetic correlation between mean and residual variance indicates the response to selection when the breeding goal is to increase uniformity. Our low estimates of $h_{v}^{2}$ and positive $r_{\mathrm{mv}}$ in M2 and M3 suggest that the response to selection may be limited if the desired response is to increase FA or CS scores and reduce their variability.

### 4.3 Implications for Breeding

Foot and leg scores have become an important part of the genetic evaluation of beef cattle worldwide because animals with undesirable proportions, foot angle, and fore and hind limbs may have problems with bones and hooves, which can affect their productive performance and reduce their longevity (Filho et al. 2010). Hoof alignment is directly related to the overall health of the animal and its longevity in the herd, as any postural irregularity can cause discomfort in different ways and degrees. In a study of dairy cows, Sewalem et al. (2004) reported that the longevity of cows with legs that were either too straight or too curved was negatively affected, resulting in a shorter productive life, even when the animals had favourable genetic potential for other economically important traits.

Based on our results, selection to simultaneously alter the mean and variance of FA and CS could be explored using index selection theory, which implies that mean and residual variance should be considered as distinct traits (Mulder et al. 2008; Ros et al. 2004). For example, if the mean of the trait is close to its optimum, a restricted index could be used to maintain the mean while reducing the residual variance. Similarly, Berghof et al. (2019) presented an alternative to include uniformity (resilience) in selection indices by using indicators based on their economic values (reduced labor and health costs). Given the importance and documented economic losses due to poor hoof structure (Boelling and Pollott 1998; Boettcher et al. 1998; Enting et al. 1997; Merks et al. 2012), including uniformity parameters in selection indices may be an appropriate approach. The authors also evaluated the inclusion of these indicators in simulated scenarios for pig and dairy cattle breeding systems. The results of these simulations indicated that the selection response was greater when the indicators were considered in selection, and therefore uniformity should be considered when calculating total merit indices of animals. Lastly, because FA and CS are recommended to be collected at different ages (covering different production periods) and are relatively easy to obtain, they can be used to calculate uniformity or resilience indicators to be evaluated genetically.

Some limitations to selection for uniformity may arise from the data structure, which can result in low genetic variance in residual variance, low heritability of residual variance and high positive genetic correlation between the mean and residual variance. An optimal data set for predicting genetic parameters from genetic heterogeneity of residual variance models should be structured to reflect the complexity of breeding programs in different environments. Given that selection and production often occur in different environments, properly accounting for environmental variation is crucial to minimise bias in variance estimates. This can be achieved by ensuring balanced environmental conditions, where data collection spans multiple environments to capture genotype‐by‐environment interactions and prevent inflation or underestimation of genetic effects. In addition, incorporating alternative methods for measuring uniformity, such as calculating standard deviations based on data structure, can improve the accuracy and interpretability of genetic variance estimates for residual variance.

In addition to refining data structure and variance modelling, advances in precision livestock farming (PLF) technologies provide an opportunity to further improve phenotypic collection. With the recent development of PLF technologies, digital data recording is another alternative to collect phenotypes more frequently, in greater quantities, and at a much faster rate (Morota et al., 2018). Phenotype collection can be laborious, costly, and prone to human error. PLF technologies, such as imaging or sensors, can be used to assess different production traits. Digital phenotyping tools are being developed to minimise lameness in pigs by evaluating locomotion and front/rear legs (Psota et al. 2020, 2019). Research on digital data recording and investigation of the inclusion of uniformity FA and CS should be explored to evaluate genetic and economic responses to breeding goals in beef cattle.

Lastly, genomic selection can potentially be used to predict uniformity parameters. The advantages of using genomic selection include increased accuracy of genetic value estimates, reduced generation intervals by identifying genetically superior animals before they express the phenotype of interest, and correcting for potential pedigree errors that would negatively affect the accuracy of estimates (Goddard et al. 2010). Additionally, genomic selection plays a role in increasing the reliability of EBV, especially for young animals. However, few studies have been published using genomic data to evaluate genetic heterogeneity of residual variance (Mulder et al. 2016; Sell‐Kubiak et al. 2015; Sae‐Lim et al. 2017), likely due to the complex task of fitting high‐dimensional genomic data using DHGLM or the genetically structured variance model and the current lack of software capable of incorporating genomic information in their evaluations. In our study, genomic data were not used because (1) the DHGLM model did not converge and (2) available software for fitting the genetically structured variance model does not yet support genomic data. Further research should be conducted to incorporate genomic data with different models to evaluate genetic heterogeneity of residual variance in economically important traits.

## 5 Conclusion

To our knowledge, this is one of the first studies of genetic heterogeneity of residual variance for foot score traits in beef cattle and to estimate genetic parameters for foot score uniformity. The results of this study suggest the presence of additive genetic effects on residual variance for FA and CS, and the possibility of altering the residual variance through selection. The genetic coefficient of variation on M2 and M3 averaged 0.07 for FA and 0.04 for CS, indicating that changing the mean of FA or CS would also change the residual variance by 7% and 4% for FA and CS, respectively.

The low heritability estimates for residual variance were within the range reported in other studies in livestock species and reinforced the need for larger datasets to more accurately estimate heritabilities for residual variance. Additionally, positive genetic correlations between means and residual variances suggest that the genetic response to direct selection may be limited, but alternatives such as the development of uniformity indicators could be used in selection indices along with other breeding goals, such as growth, reproduction, and health traits. Further studies should be conducted to evaluate the incorporation of such indicators in breeding programs and to confirm the results obtained from the exploratory approach in this study.

## Author Contributions

S.T.A., G.M. and N.I.‐E. conceived the study. S.T.A. carried out the data analyses and prepared the first draft of the paper. K.J.R., A.G., N.I.‐E. and G.M. provided technical support for the analyses, revised and edited the manuscript, and contributed to the interpretation and discussion of the results. G.M. supervised the study. All authors have read and approved the final manuscript.

## Ethics Statement

The datasets used were obtained from pre‐existing databases (American Angus Association and Angus Genetics Inc., Saint Joseph, MO, USA). Therefore, Animal Care and Use Committee approval was not required for this study.

## Consent

The authors have nothing to report.

## Conflicts of Interest

K.J.R. and A.G. were employees of Angus Genetics Inc.—American Angus Association (Saint Joseph, MO, USA). The other authors declare no conflicts of interest.

## Data Availability

The data supporting the results of this article are property of the American Angus Association cattle producers, and this information is commercially sensitive, therefore the data cannot be made available.
